# Psychological well‐being in patients with aneurysms‐osteoarthritis syndrome

**DOI:** 10.1002/ajmg.a.61209

**Published:** 2019-05-27

**Authors:** Lidia R. Bons, Allard T. van den Hoven, Ayda E. Damirchi, Denise van der Linde, Silvy Dekker, Robert M. Kauling, Ingrid M. B. H. van de Laar, Elisabeth M. W. J. Utens, Ricardo P. J. Budde, Jolien W. Roos‐Hesselink

**Affiliations:** ^1^ Department of Cardiology, Erasmus MC University Medical Center Rotterdam Rotterdam the Netherlands; ^2^ Department of Clinical Genetics, Erasmus MC University Medical Center Rotterdam Rotterdam the Netherlands; ^3^ Department of Child and Adolescent Psychiatry/Psychology, Erasmus MC University Medical Center Rotterdam Rotterdam the Netherlands; ^4^ Research Institute of Child Development and Education University of Amsterdam Amsterdam the Netherlands; ^5^ Academic Center for Child Psychiatry the Bascule/Department of Child and Adolescent Psychiatry Academic Medical Center Amsterdam the Netherlands; ^6^ Department of Radiology and Nuclear Medicine, Erasmus MC University Medical Center Rotterdam Rotterdam the Netherlands

**Keywords:** aneurysms‐osteoarthritis syndrome, anxiety, aortic disease, depression, quality of life

## Abstract

Aneurysms‐osteoarthritis syndrome (AOS) is characterized by arterial aneurysms and dissection in combination with early‐onset osteoarthritis, which can impact quality of life. We describe the subjective quality of life and investigate anxiety and depression in 28 AOS patients aged 15–73 years. Three questionnaires were used: 36‐Item Short Form Survey (SF‐36), hospital anxiety and depression scale (HADS) and Rotterdam disease specific questionnaire. Results of the SF‐36 and HADS were compared to a reference Dutch cohort and the SF‐36 questionnaire also to patients with Marfan syndrome. Compared to the general population, AOS patients scored significantly lower on the following SF‐36 domains: physical functioning, vitality, social functioning, bodily pain, and general health. Physical functioning was also lower than in Marfan patients. Patients with AOS scored higher on the HADS depression scale, while anxiety did not show a significant difference compared to the general population. No difference in SF‐36 and HADS domain scores were found between patient with and without orthopaedic symptoms and patients with or without previous aortic surgery. Additionally, we found that patients' worries for their future and heredity of their disease are important factors for anxiety, which should be addressed in clinical practice.

## 1 INTRODUCTION

An aneurysm or dilatation of the thoracic aorta can cause aortic dissection, which is a potentially life threatening event as over half of all patients with an acute thoracic aortic dissection die within 30 days (Melvinsdottir et al., 2016). In 20% of the patients an aortic aneurysm results from a heritable thoracic aortic disease (HTAD) (Caglayan & Dundar, 2009; Hannuksela, Stattin, Johansson, & Carlberg, 2015). In 2011, a new HTAD was described, the so‐called “aneurysms‐osteoarthritis syndrome (AOS),” caused by a pathogenic variant in the *SMAD3* gene (van de Laar et al., 2011), which is part of the TGF‐β pathway. Aneurysms‐osteoarthritis syndrome has many similarities with Loeys–Dietz syndrome (LDS), and is therefore also referred to as LDS type 3. In AOS, aneurysms can occur within the aorta and other arteries (among which the splenic, iliac, hepatic, and intracranial arteries). Furthermore, the arteries show tortuosity and aortic dissections or ruptures already occur in a mildly dilated aorta. In 18% of the patients aortic dissection is even the first manifestation of the disease (van der Linde et al., 2012). In addition to the vascular findings, joint abnormalities are an important feature of this syndrome, which are often the reason for first presentation. These joint abnormalities include osteoarthritis and osteochondritis dissecans at a relatively young age (van de Laar et al., 2012). Other characteristics associated with pathogenic variants in the *SMAD3* gene are widely spaced eyes, bifid uvula, umbilical or inguinal hernias varices, velvety skin, and striae (van de Laar et al., 2011). These physical symptoms and the risk of life threatening dissection of the arteries might cause reduced quality of life, anxiety, and depression. Anxiety in AOS patients can also be caused by experiencing the consequences of the disease through relatives, since this autosomal dominant genetic disorder is often diagnosed in multiple family members. Therefore knowledge about psychological well‐being and causes of impaired quality of life and anxiety or depression in AOS patients is important in order to develop specific management strategies. Although psychosocial well‐being has been investigated for other vasculopathies such as Marfan syndrome (Gritti et al., 2015) and Ehlers–Danlos (Berglund, Pettersson, Pigg, & Kristiansson, 2015), no attention has been paid yet to the quality of life and occurrence of depression or anxiety in patients with this life‐threatening syndrome. Therefore, the aim of this study was to comprehensively describe the subjective quality of life and investigate anxiety and depression in AOS patients.

## 2 MATERIALS AND METHODS

### 2.1 Study population

All carriers of a pathogenic variant in the *SMAD3* gene undergoing follow‐up in our tertiary center per in‐house protocol since January 2009 were invited for this study. Family members which were 50% risk carriers with obvious AOS related symptoms (aortic dilatation or osteoarthritis at an early age) were also included. Demographic and clinical data were obtained from the electronic patient files. Diabetes mellitus was defined as current use of medication. As part of our protocol, all patients underwent echocardiography and whole‐body computed tomography angiography (CTA). The aortic measurements of the sinus of Valsalva, ascending aorta, aortic arch, and descending aorta were measured using the inner edge‐to‐inner edge method on the most recent CTA. Aneurysms and dissections were categorized by the following locations and definition: head and neck, thoracic, coronary, abdominal, leg and/or arm or pulmonary artery. Information on the following valvular, ventricular and arrhythmic abnormalities was collected: bicuspid aortic valve, aortic stenosis (Vmax >2.5 m/s), aortic regurgitation (at least moderate) (Lancellotti et al., 2013), valvular disease other than from the aortic valve, congenital heart disorders, ventricular hypertrophy (septal wall>13 mm), left ventricular dilatation (diastolic diameter >60 mm), and atrial fibrillation (former, paroxysmal or current). The study complied with the Declaration of Helsinki and was approved by the medical ethical committee of the Erasmus Medical Centre (MEC17‐057). Written informed consent was provided by all patients.

### 2.2 Questionnaires

All participants received three questionnaires: the Short Form‐36 Health Survey (SF‐36) (Ware & Sherbourne, 1992), the hospital anxiety and depression scale (HADS) (Snaith, 2003) and the Rotterdam disease specific questionnaire. The questionnaires were sent at first on the 14th of November 2017 and were collected until the 1st of February 2018. If participants did not respond at first, they received a maximum of two reminders. The SF‐36 was used to determine patient‐reported quality of life. It covers the following eight domains: physical functioning, role limitations due to physical health, bodily pain, general health, mental health, role limitations due to mental health, vitality (energy or fatigue related), and social functioning. The scale ranges from 0 through 100 points. A lower score per subcategory reflects a lower quality of life on that life domain. In addition, two sum scores, the mental component summary (MCS) and physical component summary (PCS), were calculated (Ware JE, 1994). These summary scores are standardized according to the general Dutch population (Aaronson et al., 1998), which means that all scores above and below 50 are above and below the average in the Dutch population. The HADS questionnaire determines the levels of anxiety and depression on two subscales with a total score ranking from 1 to 21. A score of 0–7 for either subscale is in the normal range, a score of 8–10 is possible abnormal and 11 or higher indicates the probable presence of anxiety or depression. The “Rotterdam disease specific questionnaire” was developed by our multidisciplinary team in the Erasmus Medical Center (Supporting information) to investigate the impact of having AOS related aortic aneurysm on daily life, work participation, sexual functioning, pregnancy wish, and sports participation. Patients received 18 statements and were asked to grade how they felt on a continuous scale from 0 to 10, 0 being “I completely disagree” and 10 being “I completely agree”.

### 2.3 Comparison with the general population and other aortic disease patients (Marfan syndrome)

For the HADS and SF‐36 questionnaires we compared our data to the reference values of the age‐matched general Dutch population (Aaronson et al., 1998; Spinhoven et al., 1997). For the SF‐36, a cohort of age and sex matched Marfan patients was also available, which allowed us to compare AOS with another syndromic HTAD (Rand‐Hendriksen et al., 2010). For the results of the Rotterdam disease specific questionnaire, there are no reference values available yet, because this questionnaire was newly developed for this study's aim.

### 2.4 Statistical analysis

Continuous variables with a normal distribution were reported as mean with ± *SD* and the median and interquartile range was reported in case of non‐normal distribution. Categorical variables were summarized as frequencies and percentages. Data distribution was checked using histograms. Because of the non‐normally distribution of the values in the domains of the SF‐36 and HADS questionnaires, the median and interquartile ranges are presented in table 1 and the *p*‐value of the one‐sample Wilcoxon signed rank test was presented in the text and figures. With the one‐sample Wilcoxon signed rank test the median of a continuous variable in our cohort was compared with a hypothesized median of a reference group. Since our reference article showed their values only with mean ± *SD*, we assumed that the variables were distributed normally. Because mean and median are comparable in normally distributed variables, we used the mean of the reference as hypothesized median in our nonparametric test. To visually compare our data with reference data presented as mean ± *SD*, also our data were presented as mean ± *SD* in the figures although we could not proof normal distribution. However, only small differences were found between the calculated mean and median of the HADS and SF‐36 domain scores. To further investigate if patients with orthopaedic symptoms or previous aortic surgery had higher levels on the SF‐36 and HADS domains, we performed the Mann–Whitney *U* test. A *p*‐value of less than 0.05 was considered statistically significant. All statistical analyses were performed using the Statistical Package for Social Sciences (Released 2012. IBM SPSS Statistics for Windows, Version 21.0. Armonk, New York: IBM Corp).

**Table 1:** Results of SF‐36 and HADS in patients with AOS

	Domain/scales	Score	Summary measures^a^	Score
SF‐36	Physical functioning	45.0 (30.0–78.8)	PCS	34.3 (25.0–48.2)
	Role limitations due to physical health	37.5 (0.0–100.0)		
	Bodily pain	57.5 (35.0–75.0)			
	General health	40.0 (25.0–55.0)		
	Mental health	76.0 (57.0–88.0)	MCS	50.4 (39.4–59.9)
	Role limitations due to mental health	100.0 (33.3–100.0)		
	Vitality	50.0 (20.0–63.8)		
	Social functioning	62.5 (50.0–87.5)		
HADS	Depression scale	5.0 (2.0–9.8)		
	Anxiety scale	5.0 (2.0–7.8)		

*Note*: Data is shown as median (25–75%).

Abbreviations: AOS, aneurysms‐osteoarthritis syndrome; HADS, hospital anxiety and depression scale; MCS, mental component summary; PCS, physical component summary.

a Standardized scores with use of the general Dutch population (Aaronson et al., 1998).

## 3 RESULTS

### 3.1 Study population

Of the 31 patients with AOS in our center, 28 patients (90%) agreed to participate and returned the questionnaires. The other three patients were approached two times, but could not be reached (*n* = 2) or decided not to participate due to time‐constraints (*n* = 1). There were no major differences between the 28 responders and 3 nonresponders. The baseline characteristics of the 28 study patients are presented in Table 2. Our cohort contained 23 participants with a confirmed SMAD3 mutation representing 10 different genetic mutations with the most common heterozygous mutation (R287W, 859C>T, SMAD3 ex 9) present in 9 patients. The mean age was 44.0 ± 17.3 years with an age range from 15 to 73 years. Of the 28 patients, 17 (61%) were women. Cardiac or vascular abnormalities were present in 22 (79%) patients and orthopaedic symptoms were present in 24 (86%) patients. In 18 (64%) of the 28 patients both cardiovascular manifestations and orthopaedic symptoms were reported.

**Table 2:** Baseline characteristics

Baseline characteristics	AOS patients (*N* = 28)
Sex (female)	17 (61%)
Age (years)	44.0 ± 17.3
Confirmed SMAD3 mutation carriers^a^	23 (82%)
BMI (kg/m2)	24.9 ± 3.4
Smoking (currently)	2 (7%)
Systolic blood pressure (mmHg)	128.2 ± 16.9
Diastolic blood pressure (mmHg)	80.4 ± 9.5
Medication use	14 (50%)
Beta‐blocker	8 (29%)
Diuretics	0 (0%)
ACE inhibitors	4 (14%)
Angiotensin receptor blocker	2 (7%)
Calcium channel blocker	0 (0%)
Cholesterol lowering drugs (statins or other)	3 (11%)
Platelet inhibitor	6 (21%)
Oral anticoagulant	2 (7%)
Comorbidity	4 (14%)
Diabetes mellitus	0 (0%)
Coronary artery disease	1 (4%)
Aortic aneurysm or dissection	19 (68%)
History of aortic surgery	9 (32%)
Thoracic aortic aneurysms (>40 mm)^b^	5 (18%)
Head and neck arterial anomaly	6 (21%)
Coronary arterial anomaly	0 (0%)
Abdominal arterial anomaly	5 (18%)
Leg or arm arterial anomaly	0 (0%)
Pulmonary artery dilatation (>40 mm)	2 (7%)
Aortic diameter^b^	
Sinus of Valsalva	36.0 ± 3.5 (range 30–44)
Ascending aorta	30.6 ± 3.6 (range 25–40)
Aortic arch	26.5 ± 3.7 (range 21–34)
Descending aorta	24.9 ± 3.4 (range 19–33)
Cardiac anomalies	9 (32%)
Bicuspid valve	0 (0%)
Aortic stenosis (Vmax >250 m/s)	0 (0%)
Aortic regurgitation (at least moderate)	0 (0%)
Valve disease other than aortic (at least moderate)	1 (4%)^c^
Congenital heart disease (i.e., VSD)	1 (4%)
Ventricular hypertrophy (>13 mm)	2 (7%)
Left ventricular dilatation (>60 mm)	2 (7%)
Atrial fibrillation (former/paroxysmal or currently)	4 (14%)
Age first vascular or cardiac abnormalities (in years)	38.0 (26.5–56.0)
Orthopaedic abnormalities^d^	24 (86%)
Age first orthopaedic abnormalities (in years)	20.0 (13.8–46.0)

*Note*: Data is shown as median (25–75%), mean ± *SD* or as *N* (%). Missing values for BMI (*n* = 2).

a Five patients have a 50% chance of having AOS, since they are not yet genetically tested. They are included because they showed significant aortic, cardiac or orthopaedic symptoms associated with AOS.

b Aortic diameters of the sinus of Valsalva and ascending aorta and prevalence of thoracic aortic aneurysm (>40 mm) are presented for patients who have not undergone aortic surgery.

c This patients showed moderate mitral valve regurgitation.

d Orthopaedic abnormalities such as arthritis, arthrosis, osteochondritis dissecans, orthopaedic surgeries, osteosarcomas, instability of the joints, joint or muscle pain.

### 3.2 Quality of life, anxiety, and depression

**Figure 1:**
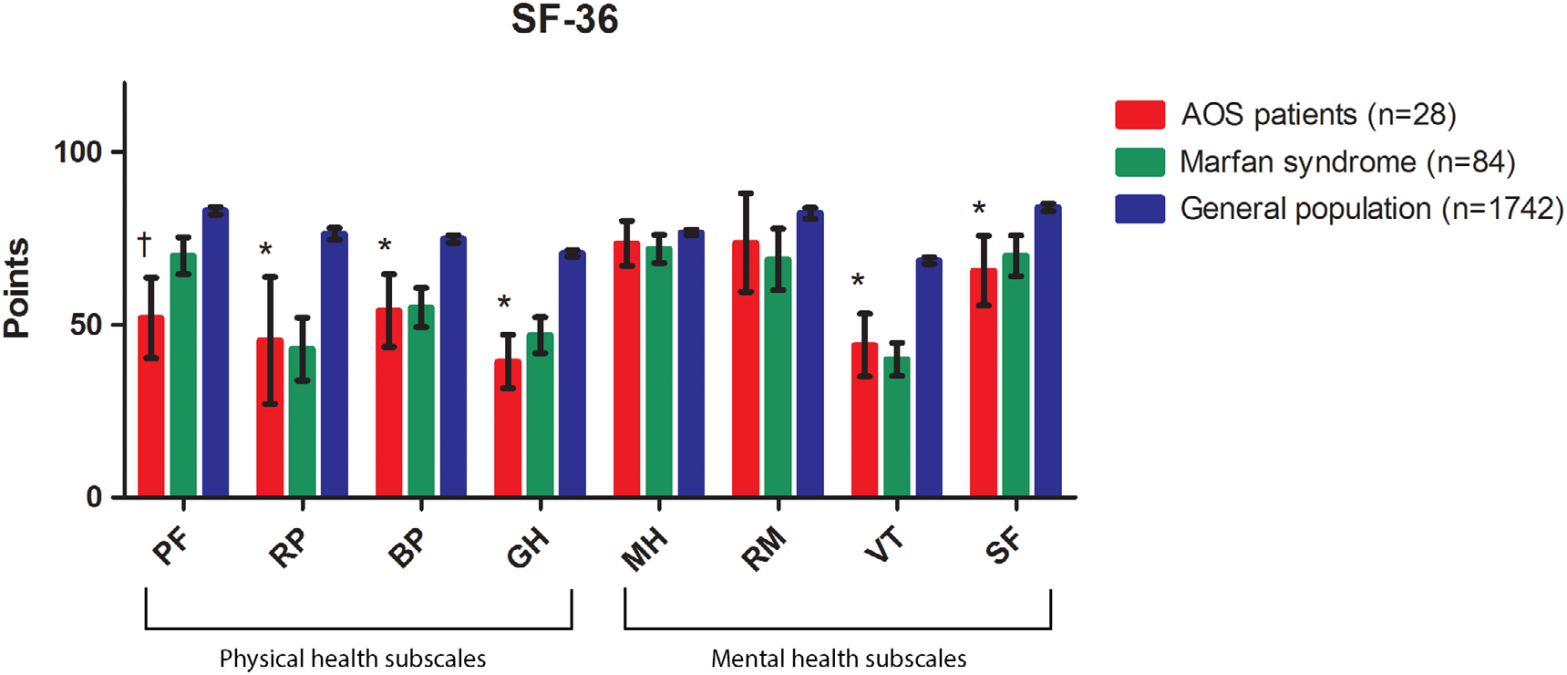
Comparison of eight SF‐36 domains between AOS patients, Marfan patients and healthy control. Data is shown as mean (incl. 95% CI). The SF‐36 scale ranges from 0 through 100 points. The lower the point count per subcategory, the more prevalent it is that the individual has a negative effect of that sub scale's premise. For social functioning and general health one patient was missing, because he forgets to fill in one page of the questionnaire. ^*^ Significant lower compared to the mean of the general population (One‐sample Wilcoxon signed rank test). ^†^ Significant lower compared to both the mean of the general population and the mean of patients with Marfan syndrome (One‐sample Wilcoxon signed rank test). AOS, aneurysms‐osteoarthritis syndrome; BP, bodily pain; GH, general health; HADS, hospital anxiety and depression scale; MH, mental health; PF, physical functioning; RM, role limitations due to mental health; RP, role limitations due to physical health; SF, social functioning; VT, vitality

The median values with interquartile range of the domains from the SF‐36 and HADS questionnaires are presented in Table 1. Our cohort scored significantly lower compared to the age‐matched reference group of 1,742 Dutch citizens (Aaronson et al., 1998) on the following domains: physical functioning (*p* < .001), role limitations physical health (*p* = .001), bodily pain (*p* = .001), general health (*p* < .001), vitality (*p* < .001), and social functioning (*p* = .002). AOS patients showed a standardized PCS score of 34.3 (25.0–48.2) and a MCS score of 50.4 (39.4–59.9). When comparing the SF‐36 with age‐matched Marfan patients, only physical health was lower in patients with AOS (*p* = .005). The mean values with standard deviation of the domains from the SF‐36 questionnaire for AOS patients, Marfan patients and the reference group are visualized in Figure 1.

**Figure 2:**
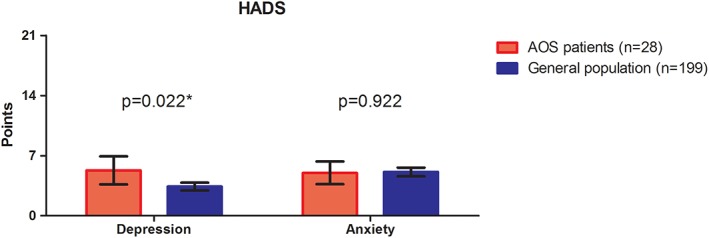
Comparison of two HADS domains between AOS patients and healthy control. Data is shown as mean (incl. 95% CI). The anxiety and depression sub scales have a point system of 0 through 21 points total. The higher the point count for any sub scale, the more likely that the individual suffers from anxiety or depression. ^*^Significant higher compared to the general population (one‐sample Wilcoxon signed rank test)

The HADS questionnaire showed no differences in anxiety (median 5.0 vs. 5.1, *p* = .569) between AOS patients and the general population. However, patients with AOS scored higher on the depression subscale (median 5.0 vs. 3.4, *p* = .036) compared to a sample of 199 Dutch adults (Spinhoven et al., 1997). In our population of AOS patients, two patients (7%) scored above the cut‐off, indicative for clinical depression, while one patient (4%) scored in the range for clinical anxiety. The mean values with standard deviation of the domains from the HADS questionnaire are shown in Figure 2 for the AOS patients and the reference group.

No significant differences in SF‐36 and HADS domain scores were found between patient with and without orthopaedic symptoms and patients with or without previous aortic surgery.

### 3.3 Disease specific anxieties/concerns

**Figure 3:**
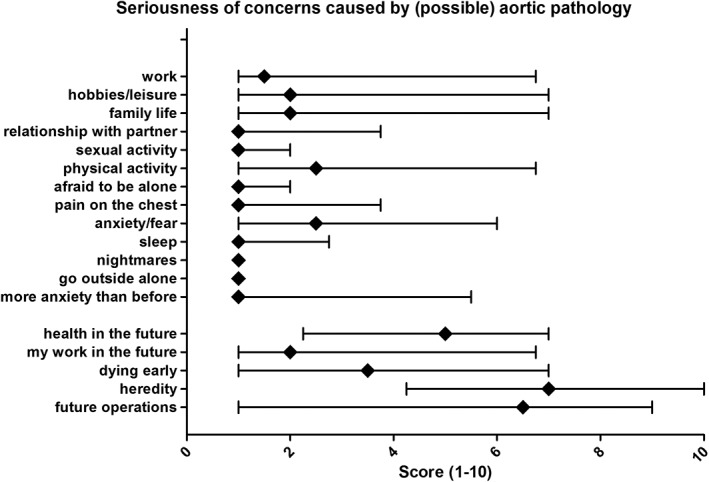
Results of the Rotterdam disease specific questionnaire. This figure shows the influence of (possible) aortic dilatation or dissection on different aspects of life. Data shown as median with interquartile range 25–75%

In the Rotterdam disease specific questionnaire, the majority of the patients reported fear and/or anxiety according to their future or the future of their siblings or offspring's (Figure 3). Concerns about dying at an early age (median 3.5, IQR 1.0–7.0), future health (median 5.0, IQR 2.3–7.0), future surgery (median 6.5, IQR 1.0–9.0) and heredity of their disease (median 7.0, IQR 4.3–10.0) were mentioned. The risk of developing aortic dilatation or dissection or already having aortic pathology did not have a significant impact on participation in work, hobbies, sexual activities, and physical activities.

## 4 DISCUSSION

The results of our study show that patients with AOS report a lower quality of life, mainly on the physical health subscales, and higher scores on the depression subscale compared to the general Dutch population. Disease specific anxiety was most often related to the future of their own health or the health of their close relatives.

Although anxiety, depression, and quality of life have been described in other patients groups with aortic pathology (Boman, Bryman, & Moller, 2004; Goldfinger et al., 2017; Winnerkvist, Brorsson, & Radegran, 2006), specifically after thoracic aortic surgery (Okamoto, Motomura, Murashima, & Takamoto, 2013), it has not yet been described in AOS patients or Loeys–Dietz syndrome. In general, AOS is not a very well‐known syndrome among cardiologist and other specialists, while pathogenic variants in the *SMAD3* gene are responsible for 2% of familial thoracic aortic disease (Regalado et al., 2011). This patient group is of great interest, because of their recently discovered extensive presentation including severe joint abnormalities and most importantly aggressive cardiovascular phenotype requiring vigilant follow‐up (van der Linde et al., 2012). Therefore it is important for all clinicians involved in the care for these patients to be aware of the psychological aspects in order to provide adequate care. We showed lower SF‐36 scores, on almost all domains compared to the general population (Aaronson et al., 1998). The median PCS score of our patients with pathogenic variants in the *SMAD3* gene was 34.3, which means a much lower physical health than the general population, while the median MCS score (50.4) showed that mental health was comparable with the general population. However, on two subscales of mental health, namely vitality and social functioning, AOS patient did score lower than the general population. We know that middle aged Dutch patients with congenital heart disease show similar or even more favorable levels compared to normative data using the SF‐36 survey (Opic et al., 2016). This difference between patients with congenital heart disease and AOS patients can be explained by the moment at which the symptoms are present. Orthopaedic and cardiovascular symptoms associated with AOS start at a median age of 22 and 38 years respectively, which cause an acute change in patients' health with clear impact on quality of life. Whereas the patient with congenital heart disease are known with their defect from birth, AOS patients need to adapt and accept that they have this disease at later age. Compared to patients with Marfan syndrome (Rand‐Hendriksen et al., 2010), AOS patients scored only lower on the SF‐36 questionnaire for the domain of physical functioning. This can be a result of the more extensive presentation of musculoskeletal complaints in patients with AOS including osteoarthritis, osteochondritis dissecans, scoliosis, and pectus excavatum (Loeys et al., 2010; van de Laar et al., 2012). This assumption is supported by data from patients with Ehlers–Danlos syndrome, a connective tissue disorder which presents with extreme musculoskeletal symptoms including hypermobility (De Paepe & Malfait, 2012). Ehlers–Danlos patients report even lower physical function score (39.6) and general health score (26.8) compared to AOS patients (Berglund et al., 2015; Bovet, Carlson, & Taylor, 2016). We did not find a difference between patients with and without orthopaedic symptoms. However, this should be tested in larger cohorts, since our cohort might be too small to prove the association between orthopaedic symptoms and reduced quality of life. In conclusion, AOS patients and probably also patients with other heritable thoracic aortic disease, have lower quality of life than the general population, but there seem to be some differences between syndromes. By assessing quality of life, anxiety, and depression, we found unfavorable outcomes and impairments, warranting attention and in some cases treatment. In our population of patients with pathogenic variants in the *SMAD3* gene, 4% scored above the cut‐off for clinical anxiety, while 7% scored in the range for clinical depression. These percentages are comparable to or even slightly lower than the prevalence's in the general German population, which are 5.2% for anxiety and 9.6% for depression based on the HADS questionnaire (Hinz & Brahler, 2011). However, the median of the continuous outcome of the depression scale was higher in OAS patients than in the general population. With the development of our own Rotterdam disease specific questionnaire, we were able to identify patients concerns due to their disease. Most importantly, patients report worries concerning the future of their own health or the health of their close relatives. These results emphasize the need for physicians to discuss patient's future and risk for family members and check in each patient whether someone is concerned about this. This is not only important for AOS, but for all inherited syndromes. Generic questionnaires such as SF‐36 or EuroQol (EQ‐5D) are commonly used for quality of life assessment. Although the use of validated questionnaires is extremely important, these questionnaires do not distinguish between quality of life based on the disease itself or as a result of other problems like small or short‐term injuries and life events. With the use of more disease‐specific questionnaires, such as the Rotterdam disease specific questionnaire, in larger cohorts we will be able to identify the cause of physiological burden in a disease more precisely in the future. Nevertheless, before using these questionnaires in clinical practice, they should be validated.

### 4.1 Limitations

In this study, we used questionnaires to measure self‐reported quality of life, anxiety, and depression, which may have caused documentation of more complaints than patients would have mentioned spontaneously. Also, it may cause some information bias, although we assume that the high response rate of 90% reduced the chance of bias. Because of our single center design, we included a small cohort, which prevented us from extensive identification of factors associated with quality of life, anxiety, or depression.

### 4.2 Conclusion

In conclusion, our population of AOS patients showed reduced quality of life in comparison with the general population on physical functioning, role limitations due to physical functioning, bodily pain, general health, vitality, and social functioning. Physical functioning was also lower than in Marfan patients. Although the prevalence of depression was similar to the general population, patients with AOS scored significantly higher on the depression scale, which physicians must be aware of to provide good medical care. Additionally, we found that patients' worries for their future and heredity of their disease are important factors for anxiety, which should be addressed in clinical practice.

## CONFLICT OF INTEREST

The authors declare no potential conflict of interest.

## Supporting information


**Appendix S1**: Supporting Information.

## References

[ajmga61209-bib-0001] Aaronson, N. K. , Muller, M. , Cohen, P. D. , Essink‐Bot, M. L. , Fekkes, M. , Sanderman, R. , … Verrips, E. (1998). Translation, validation, and norming of the Dutch language version of the SF‐36 health survey in community and chronic disease populations. Journal of Clinical Epidemiology, 51(11), 1055–1068. 9817123 10.1016/s0895-4356(98)00097-3

[ajmga61209-bib-0002] Berglund, B. , Pettersson, C. , Pigg, M. , & Kristiansson, P. (2015). Self‐reported quality of life, anxiety and depression in individuals with Ehlers‐Danlos syndrome (EDS): A questionnaire study. BMC Musculoskeletal Disorders, 16, 89. 25880527 10.1186/s12891-015-0549-7 PMC4403907

[ajmga61209-bib-0003] Boman, U. W. , Bryman, I. , & Moller, A. (2004). Psychological well‐being in women with turner syndrome: Somatic and social correlates. Journal of Psychosomatic Obstetrics and Gynaecology, 25(3–4), 211–219. 15715020 10.1080/01674820400017855

[ajmga61209-bib-0004] Bovet, C. , Carlson, M. , & Taylor, M. (2016). Quality of life, unmet needs, and iatrogenic injuries in rehabilitation of patients with Ehlers‐Danlos syndrome hypermobility type/joint hypermobility syndrome. American Journal of Medical Genetics. Part A, 170(8), 2044–2051. 10.1002/ajmg.a.37774 27273746

[ajmga61209-bib-0005] Caglayan, A. O. , & Dundar, M. (2009). Inherited diseases and syndromes leading to aortic aneurysms and dissections. European Journal of Cardio‐Thoracic Surgery, 35(6), 931–940. 19233667 10.1016/j.ejcts.2009.01.006

[ajmga61209-bib-0006] De Paepe, A. , & Malfait, F. (2012). The Ehlers‐Danlos syndrome, a disorder with many faces. Clinical Genetics, 82(1), 1–11. 22353005 10.1111/j.1399-0004.2012.01858.x

[ajmga61209-bib-0007] Goldfinger, J. Z. , Preiss, L. R. , Devereux, R. B. , Roman, M. J. , Hendershot, T. P. , Kroner, B. L. , … Gen, T. A. C. R. C. (2017). Marfan syndrome and quality of life in the GenTAC registry. Journal of the American College of Cardiology, 69(23), 2821–2830. 28595698 10.1016/j.jacc.2017.04.026 PMC5519341

[ajmga61209-bib-0008] Gritti, A. , Pisano, S. , Catone, G. , Iuliano, R. , Salvati, T. , & Gritti, P. (2015). Psychiatric and neuropsychological issues in Marfan syndrome: A critical review of the literature. International Journal of Psychiatry in Medicine, 50(4), 347–360. 26526396 10.1177/0091217415612701

[ajmga61209-bib-0009] Hannuksela, M. , Stattin, E. L. , Johansson, B. , & Carlberg, B. (2015). Screening for familial thoracic aortic aneurysms with aortic imaging does not detect all potential carriers of the disease. Aorta, 3(1), 1–8. 26798750 10.12945/j.aorta.2015.14-052 PMC4714932

[ajmga61209-bib-0010] Hinz, A. , & Brahler, E. (2011). Normative values for the hospital anxiety and depression scale (HADS) in the general German population. Journal of Psychosomatic Research, 71(2), 74–78. 21767686 10.1016/j.jpsychores.2011.01.005

[ajmga61209-bib-0011] Lancellotti, P. , Tribouilloy, C. , Hagendorff, A. , Popescu, B. A. , Edvardsen, T. , Pierard, L. A. , … Scientific Document Committee of the European Association of Cardiovascular, I . (2013). Recommendations for the echocardiographic assessment of native valvular regurgitation: An executive summary from the European Association of Cardiovascular Imaging. European Heart Journal Cardiovascular Imaging, 14(7), 611–644. 23733442 10.1093/ehjci/jet105

[ajmga61209-bib-0012] Loeys, B. L. , Dietz, H. C. , Braverman, A. C. , Callewaert, B. L. , De Backer, J. , Devereux, R. B. , … De Paepe, A. M. (2010). The revised Ghent nosology for the Marfan syndrome. Journal of Medical Genetics, 47(7), 476–485. 20591885 10.1136/jmg.2009.072785

[ajmga61209-bib-0013] Melvinsdottir, I. H. , Lund, S. H. , Agnarsson, B. A. , Sigvaldason, K. , Gudbjartsson, T. , & Geirsson, A. (2016). The incidence and mortality of acute thoracic aortic dissection: Results from a whole nation study. European Journal of Cardio‐Thoracic Surgery, 50(6), 1111–1117. 27334108 10.1093/ejcts/ezw235

[ajmga61209-bib-0014] Okamoto, Y. , Motomura, N. , Murashima, S. , & Takamoto, S. (2013). Anxiety and depression after thoracic aortic surgery or coronary artery bypass. Asian Cardiovascular & Thoracic Annals, 21(1), 22–30. 10.1177/0218492312444283 23430416

[ajmga61209-bib-0015] Opic, P. , Roos‐Hesselink, J. W. , Cuypers, J. A. , Witsenburg, M. , van den Bosch, A. , van Domburg, R. T. , … Utens, E. M. (2016). Longitudinal development of psychopathology and subjective health status in CHD adults: A 30‐ to 43‐year follow‐up in a unique cohort. Cardiology in the Young, 26(3), 547–555. 10.1017/s1047951115000700 26076871

[ajmga61209-bib-0016] Rand‐Hendriksen, S. , Johansen, H. , Semb, S. O. , Geiran, O. , Stanghelle, J. K. , & Finset, A. (2010). Health‐related quality of life in Marfan syndrome: A cross‐sectional study of short form 36 in 84 adults with a verified diagnosis. Genetics in Medicine, 12(8), 517–524. 20613543 10.1097/GIM.0b013e3181ea4c1c

[ajmga61209-bib-0017] Regalado, E. S. , Guo, D. C. , Villamizar, C. , Avidan, N. , Gilchrist, D. , McGillivray, B. , … Milewicz, D. M. (2011). Exome sequencing identifies SMAD3 mutations as a cause of familial thoracic aortic aneurysm and dissection with intracranial and other arterial aneurysms. Circulation Research, 109(6), 680–686. 21778426 10.1161/CIRCRESAHA.111.248161 PMC4115811

[ajmga61209-bib-0018] Snaith, R. P. (2003). The Hospital Anxiety And Depression Scale *Health Qual Life Outcomes* (Vol. 1, pp. 29). 10.1186/1477-7525-1-29 PMC183845 12914662

[ajmga61209-bib-0019] Spinhoven, P. , Ormel, J. , Sloekers, P. P. , Kempen, G. I. , Speckens, A. E. , & Van Hemert, A. M. (1997). A validation study of the hospital anxiety and depression scale (HADS) in different groups of Dutch subjects. Psychological Medicine, 27(2), 363–370. 9089829 10.1017/s0033291796004382

[ajmga61209-bib-0020] van de Laar, I. M. , Oldenburg, R. A. , Pals, G. , Roos‐Hesselink, J. W. , de Graaf, B. M. , Verhagen, J. M. , … Bertoli‐Avella, A. M. (2011). Mutations in SMAD3 cause a syndromic form of aortic aneurysms and dissections with early‐onset osteoarthritis. Nature Genetics, 43(2), 121–126. 21217753 10.1038/ng.744

[ajmga61209-bib-0021] van de Laar, I. M. , van der Linde, D. , Oei, E. H. , Bos, P. K. , Bessems, J. H. , Bierma‐Zeinstra, S. M. , … Wessels, M. W. (2012). Phenotypic spectrum of the SMAD3‐related aneurysms‐osteoarthritis syndrome. Journal of Medical Genetics, 49(1), 47–57. 22167769 10.1136/jmedgenet-2011-100382

[ajmga61209-bib-0022] van der Linde, D. , van de Laar, I. M. , Bertoli‐Avella, A. M. , Oldenburg, R. A. , Bekkers, J. A. , Mattace‐Raso, F. U. , … Roos‐Hesselink, J. W. (2012). Aggressive cardiovascular phenotype of aneurysms‐osteoarthritis syndrome caused by pathogenic SMAD3 variants. Journal of the American College of Cardiology, 60(5), 397–403. 22633655 10.1016/j.jacc.2011.12.052

[ajmga61209-bib-0023] Ware, J. E., Jr. , & Sherbourne, C. D. (1992). The MOS 36‐item short‐form health survey (SF‐36). I. Conceptual framework and item selection. Medical Care, 30(6), 473–483. 1593914

[ajmga61209-bib-0024] Ware, J. E. , M, K. , & Keller, S. K. (1994). SF‐36® physical and mental health summary scales: A user's manual. Boston, MA: The Health Institute.

[ajmga61209-bib-0025] Winnerkvist, A. , Brorsson, B. , & Radegran, K. (2006). Quality of life in patients with chronic type B aortic dissection. European Journal of Vascular and Endovascular Surgery, 32(1), 34–37. 16459109 10.1016/j.ejvs.2005.12.010

