# Supplementary material for: Psychological well‐being in patients with aneurysms‐osteoarthritis syndrome
Source: Am J Med Genet A. 2019 May 27;179(8):1491–7. doi: 10.1002/ajmg.a.61209 (PMC6771664; doi:10.1002/ajmg.a.61209)
Supplement: Supplementary file 1 — Appendix S1: Supporting Information. [file AJMG-179-1491-s001.docx]

**Rotterdam disease specific questionnaire**

**thoracic aortic aneurysm**

This questionnaire addresses possible limitations, consequences, concerns or anxiety that you may experience because of (the chance of) a dilatation of the aorta in the chest. Please read each question carefully and circle the number that best reflects how you felt during **the last month**.

Do not think too long about each question, as your initial response often best reflects your feelings about the subject.

The possibility, or presence, of a vascular dilatation of the aorta in my chest:

1. **Causes limitations in my work environment**

| Totally disagree | | |  | |  | |  | | Neutral | |  | |  | |  | | Totally agree | | |
| --- | --- | --- | --- | --- | --- | --- | --- | --- | --- | --- | --- | --- | --- | --- | --- | --- | --- | --- | --- |
| 1 | 2 | | 3 | | 4 | | 5 | | 6 | | 7 | | 8 | | 9 | | 10 |  |  |

1. **Causes limitations in hobby’s/ leisure activities:**

| Totally disagree | | |  | |  | |  | | Neutral | |  | |  | |  | | Totally agree | | |
| --- | --- | --- | --- | --- | --- | --- | --- | --- | --- | --- | --- | --- | --- | --- | --- | --- | --- | --- | --- |
| 1 | 2 | | 3 | | 4 | | 5 | | 6 | | 7 | | 8 | | 9 | | 10 |  |  |

1. **Has a negative influence on my family life**

| Totally disagree | | |  | |  | |  | | Neutral | |  | |  | |  | | Totally agree | | |
| --- | --- | --- | --- | --- | --- | --- | --- | --- | --- | --- | --- | --- | --- | --- | --- | --- | --- | --- | --- |
| 1 | 2 | | 3 | | 4 | | 5 | | 6 | | 7 | | 8 | | 9 | | 10 |  |  |

1. **Has a negative influence on the relationship with my partner**

| Totally disagree | | |  | |  | |  | | Neutral | |  | |  | |  | | Totally agree | | |
| --- | --- | --- | --- | --- | --- | --- | --- | --- | --- | --- | --- | --- | --- | --- | --- | --- | --- | --- | --- |
| 1 | 2 | | 3 | | 4 | | 5 | | 6 | | 7 | | 8 | | 9 | | 10 |  |  |

The possibility, or presence, of a vascular dilatation of the aorta in my chest:

1. **Causes limitations in my sexual functioning**

| Totally disagree | | |  | |  | |  | | Neutral | |  | |  | |  | | Totally agree | | |
| --- | --- | --- | --- | --- | --- | --- | --- | --- | --- | --- | --- | --- | --- | --- | --- | --- | --- | --- | --- |
| 1 | 2 | | 3 | | 4 | | 5 | | 6 | | 7 | | 8 | | 9 | | 10 |  |  |

1. **Causes avoidance of physical activities**

| Totally disagree | | |  | |  | |  | | Neutral | |  | |  | |  | | Totally agree | | |
| --- | --- | --- | --- | --- | --- | --- | --- | --- | --- | --- | --- | --- | --- | --- | --- | --- | --- | --- | --- |
| 1 | 2 | | 3 | | 4 | | 5 | | 6 | | 7 | | 8 | | 9 | | 10 |  |  |

1. **Causes anxiety to be alone**

| Totally disagree | | |  | |  | |  | | Neutral | |  | |  | |  | | Totally agree | | |
| --- | --- | --- | --- | --- | --- | --- | --- | --- | --- | --- | --- | --- | --- | --- | --- | --- | --- | --- | --- |
| 1 | 2 | | 3 | | 4 | | 5 | | 6 | | 7 | | 8 | | 9 | | 10 |  |  |

1. **Causes chest pain**

| Totally disagree | | |  | |  | |  | | Neutral | |  | |  | |  | | Totally agree | | |
| --- | --- | --- | --- | --- | --- | --- | --- | --- | --- | --- | --- | --- | --- | --- | --- | --- | --- | --- | --- |
| 1 | 2 | | 3 | | 4 | | 5 | | 6 | | 7 | | 8 | | 9 | | 10 |  |  |

1. **Causes worries/anxiety**

| Totally disagree | | |  | |  | |  | | Neutral | |  | |  | |  | | Totally agree | | |
| --- | --- | --- | --- | --- | --- | --- | --- | --- | --- | --- | --- | --- | --- | --- | --- | --- | --- | --- | --- |
| 1 | 2 | | 3 | | 4 | | 5 | | 6 | | 7 | | 8 | | 9 | | 10 |  |  |
|  |  | |  | |  | |  | |  | |  | |  | |  | |  |  |  |

1. **Causes insomnia (related to stress/worrying/anxiety)**

| Totally disagree | | |  | |  | |  | | Neutral | |  | |  | |  | | Totally agree | | |
| --- | --- | --- | --- | --- | --- | --- | --- | --- | --- | --- | --- | --- | --- | --- | --- | --- | --- | --- | --- |
| 1 | 2 | | 3 | | 4 | | 5 | | 6 | | 7 | | 8 | | 9 | | 10 |  |  |

The possibility, or presence, of a vascular dilatation of the aorta in my chest:

1. **Causes nightmares**

| Totally disagree | | |  | |  | |  | | Neutral | |  | |  | |  | | Totally agree | | |
| --- | --- | --- | --- | --- | --- | --- | --- | --- | --- | --- | --- | --- | --- | --- | --- | --- | --- | --- | --- |
| 1 | 2 | | 3 | | 4 | | 5 | | 6 | | 7 | | 8 | | 9 | | 10 |  |  |

1. **Causes avoidance of travelling alone**

| Totally disagree | | |  | |  | |  | | Neutral | |  | |  | |  | | Totally agree | | |
| --- | --- | --- | --- | --- | --- | --- | --- | --- | --- | --- | --- | --- | --- | --- | --- | --- | --- | --- | --- |
| 1 | 2 | | 3 | | 4 | | 5 | | 6 | | 7 | | 8 | | 9 | | 10 |  |  |

1. **Causes more anxiety than prior**

| Totally disagree | | |  | |  | |  | | Neutral | |  | |  | |  | | Totally agree | | |
| --- | --- | --- | --- | --- | --- | --- | --- | --- | --- | --- | --- | --- | --- | --- | --- | --- | --- | --- | --- |
| 1 | 2 | | 3 | | 4 | | 5 | | 6 | | 7 | | 8 | | 9 | | 10 |  |  |

**Because of the possibility, or presence, of a vascular dilatation:**

1. **I worry about my health in the future**

| Totally disagree | | |  | |  | |  | | Neutral | |  | |  | |  | | Totally agree | | |
| --- | --- | --- | --- | --- | --- | --- | --- | --- | --- | --- | --- | --- | --- | --- | --- | --- | --- | --- | --- |
| 1 | 2 | | 3 | | 4 | | 5 | | 6 | | 7 | | 8 | | 9 | | 10 |  |  |

1. **I worry about my work situation in de future**

| Totally disagree | | |  | |  | |  | | Neutral | |  | |  | |  | | Totally agree | | |
| --- | --- | --- | --- | --- | --- | --- | --- | --- | --- | --- | --- | --- | --- | --- | --- | --- | --- | --- | --- |
| 1 | 2 | | 3 | | 4 | | 5 | | 6 | | 7 | | 8 | | 9 | | 10 |  |  |

1. **I worry that I die prematurely**

| Totally disagree | | |  | |  | |  | | Neutral | |  | |  | |  | | Totally agree | | |
| --- | --- | --- | --- | --- | --- | --- | --- | --- | --- | --- | --- | --- | --- | --- | --- | --- | --- | --- | --- |
| 1 | 2 | | 3 | | 4 | | 5 | | 6 | | 7 | | 8 | | 9 | | 10 |  |  |

1. **I worry about the heritability of an aortic dilatation (whether my children may develop aortic dilatation)**

| Totally disagree | | |  | |  | |  | | Neutral | |  | |  | |  | | Totally agree | | |
| --- | --- | --- | --- | --- | --- | --- | --- | --- | --- | --- | --- | --- | --- | --- | --- | --- | --- | --- | --- |
| 1 | 2 | | 3 | | 4 | | 5 | | 6 | | 7 | | 8 | | 9 | | 10 |  |  |

1. **I am concerned about surgery because of an aortic dilatation**

| Totally disagree | | |  | |  | |  | | Neutral | |  | |  | |  | | Totally agree | | |
| --- | --- | --- | --- | --- | --- | --- | --- | --- | --- | --- | --- | --- | --- | --- | --- | --- | --- | --- | --- |
| 1 | 2 | | 3 | | 4 | | 5 | | 6 | | 7 | | 8 | | 9 | | 10 |  |  |

1. **Are there any other factors that negatively affect the quality of life due to the possibility, or presence, of an aortic dilatation that have not been addressed in the questions above?**

**………………………………………………………………………………………………………………………………………………………………………………………………………………………………………………………………………………………………………………………………………………………………………………………………………………………………………………………………………………………………………………………………………………………………………………………………………………………………………………………………………………………………………………………………………………………………………………………………**

1. **Has living with the possibility, or presence, of an aortic dilatation brought you something good?**

**(eg. the realization of what is important in life)**

**………………………………………………………………………………………………………………………………………………………………………………………………………………………………………………………………………………………………………………………………………………………………………………………………………………………………………………………………………………………………………………………………………………………………………………………………………………………………………………………………………………………………………………………………………………………………………………………………**

Thank you for your time!
